# Exploring the Association Between Antibiotic Prescribing Patterns and Clinical Variations in a Network of Pediatricians

**DOI:** 10.7759/cureus.19464

**Published:** 2021-11-11

**Authors:** Jordan A Nelson, Patrick K Hunter

**Affiliations:** 1 Pediatrics, University of Central Florida College of Medicine, Orlando, USA; 2 Pediatrics, Nemours Children's Health System, Orlando, USA

**Keywords:** physician behavior, pediatrics, pediatric antibiotic prescription, antibiotic overuse, clinical variation

## Abstract

Introduction: Variation in practice patterns among physicians is well-documented despite professional guidelines and increasingly uniform medical training. Variations may lead to improper utilization of healthcare resources, misdiagnosis, overdiagnosis, unnecessary treatments, and forgoing of needed interventions. One area of clinical variation and overuse of particular interest is the prescribing of antibiotics, which can lead to eventual antibiotic resistance and other negative consequences. Variations in antibiotic prescribing along with other practice patterns have been studied previously but no attempt has been made to examine the correlation between multiple practice patterns. The purpose of this study was to determine if a correlation existed between the provider behaviors studied.

Methods: A small area network of 39 pediatric providers was analyzed to determine if antibiotic prescription percentages varied. Antibiotic prescription percentages were further broken down by visit type (sick versus well). Two other practice measures, in-office lab utilization and diagnoses of food and drug allergies, were then analyzed. Data were explored primarily with Spearman’s correlation tests.

Results: Strong positive correlation was seen between a provider's antibiotic prescription percentage at sick and well visits. Strong positive correlation was seen between the antibiotic prescribing percentage and the number of in-office labs ordered. Moderate positive correlation was seen between antibiotic prescribing percentage and the percentage of a provider’s empaneled patients with any allergy diagnosis (medication, food, or seasonal).

Conclusion: This retrospective study demonstrates that variation in provider practice patterns continues to exist despite established practice guidelines from national organizations. It also demonstrates a linear correlation between multiple provider behaviors that have not previously been explored together. The presence of a correlation between clinical behaviors may suggest an underlying practice philosophy and present an opportunity for personalized, provider-specific education and quality improvement.

## Introduction

Variation in practice patterns and behaviors among physicians is documented in the scientific literature. Differences in the frequency of tonsillectomies performed were noted as early as the 1930s by Dr. J. Alison Glover [1]. These variations cannot be accounted for by medical indication alone, and 40 years after Glover’s pioneering work, a study by Bloor et al. suggested that differences in the geographical incidence of operations on the tonsils and/or adenoids are created by differences in physician practice patterns and medical opinion rather than being the result of differences in disease incidence [2,3]. A more recent study has shown that despite more uniform and advanced medical training and professional guidelines, variations still exist [4]. 

These variations affect the quality of care and contribute to healthcare disparity, especially when they lead to the overuse of diagnostic tools and treatment. Overdiagnosis is considered to occur when people are labeled with or treated for a disease that would never cause them harm [5]. This often results from unnecessary screening and can lead to the overuse of additional tests and treatments. Many reasons have been proposed for the increase in diagnostic intensity. Potential causes include the expansion of disease definitions (allowing more people to be labeled as “sick”), financial incentives within the hospital system, defensive medicine from fear of litigation, and the influence of increasingly sensitive tests that lead to the detection of minor abnormalities [5]. 

The overprescribing of antibiotic therapies to infants and children is one example of this overuse. Many guidelines for the diagnosis of bacterial illness and the use of antibiotic therapy exist. Some examples of these include the Infectious Diseases Society of America (IDSA) guidelines for the management of community-acquired pneumonia in infants and children older than three months of age [6], the American Academy of Pediatrics (AAP) clinical practice guideline for the diagnosis and management of acute otitis media [7], and the AAP clinical report on the principles of judicious antibiotic prescribing for upper respiratory tract infections in pediatrics [8]. Despite these clearly defined guidelines, many physicians continue to prescribe unnecessary antibiotics. One study found that greater than one in five pediatric care visits resulted in an antibiotic prescription and that the use of broad-spectrum antibiotics was highest among respiratory conditions for which antibiotics are typically not indicated [9]. However, this trend of over-prescription is not found among all physicians equally. In another study, standardized broad-spectrum prescribing percentages ranged from 15% to 57% across practices [10].

While the benefits of antibiotic therapies are obvious, their overuse can lead to resistance and increases in both the length and severity of the disease. Antibiotic resistance continues to evolve and represents a growing danger to all populations, including children. Resistance, however, is not the only concern with excessive or unnecessary antibiotic prescription. A 2019 study found that all commonly prescribed antibiotics during infancy are associated with subsequent diagnosis of allergic disease, and that administration of more than one class of antibiotic was associated with increased risk [11].

Antibiotic prescription is not the only area of pediatric care where overuse occurs. Excessive and unnecessary ordering of imaging, laboratory tests, and other diagnostic and screening services has been shown [12]. Using peer-reviewed literature and clinical practice guidelines, many “low-value” pediatric services have been identified. A “low-value” pediatric service is one that does not improve the child’s health or could potentially cause more harm than good [13]. Overuse leads to poor utilization of resources, medication side effects, the financial burden for patients, and other unwanted consequences. 

All these behaviors have been previously studied in isolation but analyzing various behaviors for correlations may yield insights not previously recognized. It was hypothesized that a provider’s antibiotic prescription behaviors, one of the most common and routine practices in outpatient pediatric offices, might serve as a marker for other clinical behaviors. A correlation between behaviors may suggest an underlying practice philosophy that guides the provider’s decision-making. A provider with a low threshold for prescribing antibiotics may also have a lower threshold when it comes to using in-office labs or diagnosing a child with an allergy.

To this end, a retrospective review of electronic medical records of the patients empaneled to providers within a pediatric primary care network was conducted. The primary objectives of this study were: (A) To determine whether a variation is present regarding the percentage of visits with an antibiotic prescription among the outpatient providers in a pediatric primary care small area network; (B) To determine if a correlation exists between a provider’s percentage of visits with an antibiotic prescription and the percentage of visits where an in-office lab was ordered; (C) To determine if a correlation exists between a provider’s percentage of visits with an antibiotic prescription and allergy diagnoses among a provider’s empaneled patients.

## Materials and methods

The population in this study included general pediatricians and nurse practitioners (NPs) who are employees of the pediatric primary care network. All data used in this research was extracted from the electronic medical records system using Research Electronic Data Capture (REDCap) data collection software. For the period of January 1 to December 31, 2019, data were collected on the total number of patient visits, the total number of well visits, and the total number of sick visits for each provider that meets the inclusion criteria. A well visit was defined as any visit with the primary goal is preventative care, which can include a review of health history and medical history, counseling about ways to improve health, a physical exam tailored to preventative care needs, immunizations, and screening tests if needed. A sick visit was defined as any visit focused primarily on an acute health issue arising outside of a normal preventative health schedule including treatment, symptom management, and education regarding home care.

To be included in this study, providers must have conducted their practice at pediatric outpatient clinics within the pediatric primary care network. Those who work shifts in urgent care centers in addition to outpatient clinics were included, but visits conducted at urgent care centers were not included. To be included in the study, providers must have been employed for the entire 2019 calendar year. Only visits conducted at primary care locations were included. The sample size was calculated using the G*Power software. Data on antibiotic prescriptions, in-office labs, and allergy diagnoses were also obtained. Data were explored primarily with Spearman’s correlation tests by using IBM SPSS Statistics for Windows, Version 26.0 (Released 2019. IBM Corp., Armonk, New York). Statistical significance was set at p<0.05.

For each provider, the percentage of all visits at which an antibiotic was prescribed, the percentage of well visits at which an antibiotic was prescribed, and the percentage of sick visits at which an antibiotic was prescribed were analyzed. Data were standardized by dividing the number of visits at which an antibiotic was prescribed by the total number of visits by type (total visits, well visits, and sick visits). Any dose, suspension, brand name, or generic version of antibiotic were included. Data on appropriate use, unnecessary use, escalated use of extended-spectrum antibiotics, incorrect spectrum of antibiotics, and combined antibiotic use was not examined in this study, although this could be an area for future research.

In-office laboratory utilization data were standardized by dividing the total number of each laboratory test a provider ordered by the total number of visits for that provider. The six most ordered tests among all providers were included in the analysis. Laboratory tests that were included in the analysis were the influenza test, rapid strep test, respiratory syncytial virus (RSV) test, mononucleosis test, urinalysis, and the blood glucose test. Because each test may be ordered by the provider in a variety of ways, data were extracted for all possible ordering codes.

Data on allergy diagnoses for each provider were extracted as the total number of empaneled patients with at least one allergy diagnosis. The data were standardized by dividing the total number of patients with at least one allergy diagnosis by the total number of empaneled patients per provider.

## Results

Data were gathered on 33 physicians and six NPs who met the inclusion criteria. Provider data were de-identified and provider names were replaced with a three-letter code. After examining the data for outliers, one physician was excluded due to a low number of total and well visits. The mean number of total visits per provider was 3257 with a standard deviation of 997. Eight providers (six NPs, two physicians) were excluded from the allergy and vaccine portion of the study due to a lack of empaneled patients.

Variation in the percentage of visits where an antibiotic was prescribed

**Figure 1:**
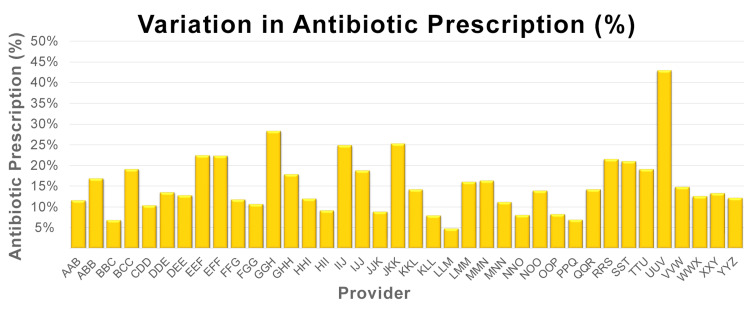
Variation in Antibiotic Prescription (%)

The mean percentage of all visits where an antibiotic was prescribed was 15.3% with a standard deviation of 7.3%. A high variation existed in the percentage of total visits at which an antibiotic was prescribed, with antibiotic prescription percentages as high as 42.9% and as low as 4.8% observed (Figure 1)

**Figure 2:**
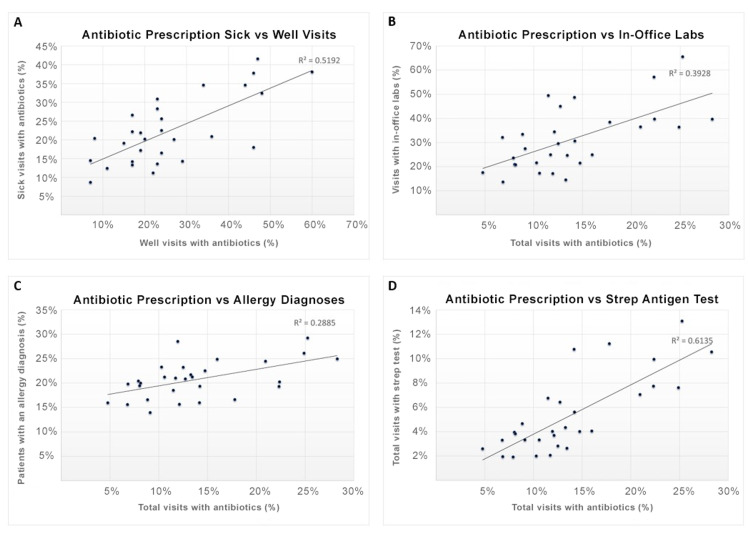
Antibiotic Prescription Correlations

Variation was also present when visits were broken down by visit type. Antibiotic prescription correlations are shown in Figure 2. The mean percentage of well visits where an antibiotic was prescribed was 2.9% with a standard deviation of 1.4%, with a high of 6% and a low of 0.7%. Among sick visits, the variation was much higher, with a mean percentage of 23.8%, a standard deviation of 9.4%, a high of 52.7%, and a low of 8.7%. Spearman’s rank-order correlation showed a strong correlation (rs = 0.677, p<0.01) between a provider’s antibiotic prescription percentage at well visits and his or her prescription percentage at sick visits (Figure 2A).

Correlation between antibiotic prescribing percentage and in-office lab use

To account for differences in the total number of patient visits among providers, the sum of all labs ordered was divided by the total number of visits for each provider. Strong positive correlation (rs = 0.605, p<0.01) was found between the percentage of total visits at which a provider prescribed an antibiotic and the number of labs ordered. When separated by visit type, the strength of the correlation increased slightly (rs = .644, p<0.01) when lab use was compared against the percentage of sick visits where an antibiotic was prescribed (Figure 2B). Among the most ordered labs, the rapid strep antigen test, rapid mononucleosis test, and urinalysis showed the most correlation with antibiotic prescribing percentage (Table 1) (Figure 2D).

**Table 1:** Correlation Between Most Ordered Labs and Total Antibiotic Prescribing Percentage

Most Ordered In-Office Labs	Spearman’s Rank-Order Correlation
Rapid Strep Antigen Test	r_s_ = 0.767, p<0.01
Rapid Mononucleosis Test	r_s_ = 0.609, p<0.01
Urinalysis	r_s_ = 0.508, p<0.01

A moderate correlation was found between antibiotic prescribing percentage and influenza tests (rs = .366, p<0.05). Correlation results between antibiotic prescription, RSV, and glucose were not significant (p>0.05).

Correlation between antibiotic prescribing percentage and allergy diagnoses

Allergy diagnosis data were analyzed for all the providers with empaneled patients (30 total). Data were standardized by dividing the total number of a provider’s empaneled patients with an allergy diagnosis by that provider's total number of empaneled patients. Moderate positive correlation (rs = 0.500, p<0.01) was found between the percentage of total visits at which a provider prescribed an antibiotic and the percentage of empaneled patients with an allergy diagnosis (Figure 2C).

## Discussion

As hypothesized, large variation was found in pediatric primary care antibiotic prescription percentage at total visits, with a difference of 38.1% between the highest and lowest prescribers. An even larger variation of 44% was observed when a patient was seen for a sick visit. Despite many providers practicing in the same geographic area - and even the same clinic - variation was still observed. When antibiotic prescription percentage was separated by visit type, a strong positive correlation was seen between well and sick visits. This finding suggests that, regardless of the reason for an appointment, certain providers may be more inclined to prescribe an antibiotic to their patients.

After establishing the presence of variation in antibiotic prescribing patterns, in-office lab ordering data were analyzed. Like antibiotic prescription, lab utilization is a marker of provider practice behavior and should not vary greatly from one provider to another. However, variation was present here as well, with lab-utilization percentages ranging from 13.7% to 57.1% of a provider’s total visits. When analyzed using Spearman’s correlation test, a strong positive correlation existed between the antibiotic prescribing percentage and the ordering of in-office labs. Providers that were higher utilizers of antibiotics were also those that more frequently ordered labs for their patients.

Lastly, the data related to allergy diagnoses among providers were analyzed. While antibiotic prescription and lab orders are both considered provider behaviors, allergy diagnosis is a patient outcome. Wide variation should not exist in the percent of empaneled patients with allergies among different providers. However, a range of 13.9% to 29.2% was seen in allergy diagnoses among providers’ empaneled patients. Additionally, a moderate correlation was seen between antibiotic prescribing percentage and the percentage of a provider’s empaneled patients with an allergy diagnosis in their medical records.

Limitations

A weakness of this study is the small sample size consisting of only one general pediatric provider network. Future studies that expand this work to include larger care networks across many regions may yield additional information as to whether location impacts a provider’s practice philosophy. Additionally, while data on antibiotic prescription percentages were extracted from the electronic medical record, the study did not differentiate between appropriate use, unnecessary use, escalated use of extended-spectrum antibiotics, incorrect spectrum of antibiotics, or combined antibiotic use.

## Conclusions

Large variation in the percentage of visits at which providers prescribed an antibiotic was found in a small pediatric primary care network. This variation persisted regardless of the visit type (sick versus well). Even though many providers worked with the same population and in the same clinic, variation in practice patterns was evident. Positive correlation existed between a provider's percentage of visits with an antibiotic prescription and the percentage of visits with at least one in-office lab ordered. Positive correlation was also found between a provider's percentage of visits with an antibiotic prescription and the percentage of his or her empaneled patients with at least one allergy diagnosis (food, drug, or seasonal) on their medical record.

In summary, providers who were high prescribers of antibiotics were, in general, more likely to be high utilizers of in-office labs and their empaneled patients were more likely to have an allergy diagnosis on their chart. The opposite was also seen - those providers who were low prescribers of antibiotics were, in general, less likely to be high utilizers of in-office labs and their empaneled patients were less likely to have an allergy diagnosis on their chart. This data suggests an underlying practice philosophy that accounts for these correlations and may present an opportunity for quality improvement.

Quality improvement measures might take a general approach. However, the data presented here and its ability to show correlations and comparisons with peers may provide an opportunity for individualized insight and education, the ultimate goals being improved patient care and judicious utilization of healthcare resources. Future analysis might look at provider behavior and correlations between specialty referral patterns, utilization of imaging, and utilization of reference laboratories. Additional studies looking at provider variables (training, age, years in practice, geographic location, compensation models, etc.) might give further insight.
